# Using machine learning models for cuffless blood pressure estimation with ballistocardiogram and impedance plethysmogram

**DOI:** 10.3389/fdgth.2025.1511667

**Published:** 2025-02-21

**Authors:** Shing-Hong Liu, Yao Sun, Bo-Yan Wu, Wenxi Chen, Xin Zhu

**Affiliations:** ^1^ Department of Computer Science and Information Engineering, Chaoyang University of Technology, Taichung City, Taiwan; ^2^ Division of Information Systems, School of Computer Science and Engineering, The University of Aizu, Aizu-Wakamatsu, Japan; ^3^ Department of AI Technology Development, M&D Data Science Center, Institute of Integrated Research, Institute of Science Tokyo, Tokyo, Japan

**Keywords:** cuffless blood pressure measurement, ballistocardiogram, impedance plethysmogram, signal quality classification, blood pressure estimation

## Abstract

**Introduction:**

Blood pressure (BP) serves as a crucial parameter in the management of three prevalent chronic diseases, hypertension, cardiovascular diseases, and cerebrovascular diseases. However, the conventional sphygmomanometer, utilizing a cuff, is unsuitable for the approach of mobile health (mHealth).

**Methods:**

Cuffless blood pressure measurement, which eliminates the need for a cuff, is considered a promising avenue. This method is based on the relationship between pulse arrival time (PAT) parameters and BP. In this study, pulse transit time (PTT) was derived from ballistocardiograms (BCG) and impedance plethysmograms (IPG) obtained from a weight-fat scale. This study aims to address two challenges using deep learning and machine learning technologies: first, identifying BCG and IPG signals with good quality, and then extracting PTT parameters from them to estimate BP. A stacked model comprising a one-dimensional convolutional neural network (1D CNN) and gated recurrent unit (GRU) was proposed to classify the quality of BCG and IPG signals. Seven parameters, including calibration-based and calibration-free PTT parameters and heart rate (HR), were examined to estimate BP using random forest (RF) and XGBoost models. Seventeen healthy subjects participated in the study, with their BP elevated through exercise. A digital sphygmomanometer was employed to measure BP as reference values. Our methodology was validated using data collected from our custom-made device.

**Results:**

The results demonstrated a signal quality classification accuracy of 0.989. Furthermore, in the five-fold cross-validation, Pearson correlation coefficients of 0.953 ± 0.007 and 0.935 ± 0.007 were achieved for systolic BP (SBP) and diastolic BP (DBP) estimations, respectively. The mean absolute differences (MADs) of XGBoost model were calculated as 3.54 ± 0.34 and 2.57 ± 0.17 mmHg for SBP and DBP, respectively.

**Discussion:**

The proposed method significantly improved the accuracy of cuffless BP measurement, indicating its potential integration into weight-fat scales as an unconstrained device for effective utilization in mHealth applications.

## 1 Introduction

The World Health Organization's Global Observatory defines mobile health (mHealth) as “medical and public health practices supported by mobile devices, such as mobile phones, patient monitoring devices, personal digital assistants, and other wireless devices” (1). mHealth addresses various challenges in healthcare services, including monitoring chronic conditions, reducing costs, empowering patients and families to manage daily healthcare, and providing direct access to health services regardless of time and location (2). mHealth systems have the potential to significantly impact healthcare by enhancing monitoring and alerting systems, facilitating clinical data collection, and maintaining records (3). Over the past decade, numerous studies have focused on developing innovative wearable devices to monitor the physiological parameters of patients with chronic diseases such as hypertension, hyperglycemia, and hyperlipidemia. These parameters generally include blood pressure (BP) and weight, and they should ideally be measured daily. Additionally, dietary services can play a role in controlling weight and BP (4–7). Previous research has shown that managing body weight can effectively lower blood pressure in hypertensive patients (8, 9). While companies like Texas Instruments and Analog Devices have developed chips to support weight-fat scales (10, 11), traditional sphygmomanometers cannot be developed as a system-on-chip (SoC) integration due to their reliance on cuffs and mechanical components. Consequently, cuffless BP measurement holds the benefit for SoC development. Some prototypes of earphones and watches have already incorporated blood pressure measurement functionalities (12, 13).

Sharwood-Smith et al. utilized electrocardiogram (ECG) and photoplethysmogram (PPG) to continuously estimate systolic blood pressure (BP) during anesthesia (14). This method relies on the pulse arrival time (PAT) measured from the R wave of ECG (proximal reference) and the peak wave of PPG (distal reference), which correlates with BP according to the Moens-Korteweg equation (15). Ballistocardiogram (BCG) waves are generated by gradients in ascending and descending aortic BP (16). The head-foot BCG can be measured using a strain gauge placed at the footplate or an accelerometer placed on the extremities (17). Kim et al. used BCG as the proximal timing reference for pulse transit time (PTT) (18). BCGs, reflecting the variations of aortic BP, can influence PTT through the amplitudes and intervals of BCGs (19). Seok et al. utilized two-channel BCGs measured from a chair and applied deep learning techniques to estimate BP (20). Martin et al. employed BCG measured from a bathroom weight scale along with PPG to estimate BP (21). Shin et al. employed BCG and PPG measured from a wrist and a finger sensor, respectively, and incorporated ECG for BP assessment (22). Liu et al. also put a bathroom weight scale and PPG sensor on the toes to measure PTT for the cuffless BP measurement (23).

Impedance plethysmography (IPG) is a technique that measures the ionic conduction of a specific body segment according to Ohm's law during cardiac contractions and relaxations (24, 25). Transient and static electrical conductivities are influenced by the dynamic and balanced conditions of arteriovenous blood volume within the body segment. In PTT measurements, wrist IPG and finger PPG were used as proximal and distal references (26), while arm IPG was also a distal reference for PAT measurement (27). Liu et al. measured BCG and IPG signals from a weight-fat scale to estimate blood pressure while individuals stood on it (28).

However, a drawback of using BCG and IPG signals from weight-fat scales is the potential signal quality issue. Inan et al. analyzed the signal-to-noise ratios (SNRs) of ECG, BCG, and IPG signals, finding SNRs of 15.8, 7.6, and 10.7 dB, respectively (29). This study showed a potential problem, how to get good quality signals when using BCG and IPG for the cuffless blood pressure measurement. The traditional method for determining physiological signal quality relies on manual markings by experts (30). However, the spatial and temporal characteristics of physiological signals can vary with factors such as age (31), mental condition (32), and sensor positions (33). Liu et al. employed two-dimensional convolutional neural networks (2D CNNs) to classify the quality of PPG signals for measuring left ventricular ejection time (34). However, PPG signals should be transformed into an image. Shin utilized 1D CNN to evaluate the signal quality of PPG (35). The advantage of CNN is that handcrafted features from signals are unnecessary but its disadvantage is the larger number of model parameters. When using machine learning (ML) for the classification of signal quality, PPG signals should be processed to extract the handcrafted features (35, 36). Prasun et al. used seven time-frequency features extracted from PPG to perform the signal quality assessment (37). Roh and Shin converted PPG to a two-dimensional phase space trajectory image using a recurrence plot and classified the signal quality (38). Because PTT is determined from BCG and IPG, the signal quality of both signals should be good for accurate measurement. Therefore, how to confirm the signal quality of synchronous BCG and IPG has the potential requirement for the cuffless BP measurement.

Pandit et al. reviewed the promise and challenge of cuffless BP measurement and proposed ML technologies to improve the accuracy (39, 40). ML has gained widespread popularity for addressing the cuffless BP measurement (37, 41–44). Feature engineering is a crucial aspect directly impacting both model performance and size. The accuracy of features significantly influences performance, with more precise features leading to improved performance and smaller model sizes. Liu et al. used ECG and PPG recorded by smartwatches to perform the cuffless BP measurement (43). They found that the calibration-based model had better performance than that of a calibration-free model. Huang et al. utilized the mixer multilayer perceptron neural network for the BP estimation with ECG and PPG signals (44). Therefore, PTT parameters extracted from BCG and IPG signals could be used with ML to improve the accuracy of BP measurement.

As previously mentioned, cuffless blood pressure measurement using BCG and IPG encounters two main obstacles. Firstly, obtaining signals of high quality is essential. Secondly, the accuracy of linear regression models is limited. This study aims to address these issues using the stacking CNN to classify the signal qualities of BCG and IPG obtained from a weight-fat scale and ensemble ML models to enhance the accuracy of cuffless BP measurement. To tackle the signal quality problem, we extracted PAT from ECG, and PPG and differential PPG (DPPG) measured at the finger as the reference. Signal quality was categorized based on the differential ratios of PAT estimated by the reference method and PTT proposed method. A stacked CNN + gated recurrent unit (GRU) was developed for signal quality classification, utilizing time series data from BCG, IPG, and differential IPG (DIPG) as input. Then, BCG is generated by heart contraction as the proximal reference, and IPG of lower limbs was the distal reference. In this study, we defined PTT as the delay time between J-wave of BCG and foot of IPG. Subsequently, six calibration-based and calibration-free PTT parameters and heart rate (HR) were extracted from these high-quality signals. We used random forest (RF) and XGBoost models to estimate BP, respectively. Seventeen subjects participated in the study, with BP measured by a digital sphygmomanometer as a reference. The results demonstrated that the performance of the proposed method significantly surpassed those of previous studies, such as the work conducted by Liu et al. (28).

## 2 Method and material

**Figure 1:**
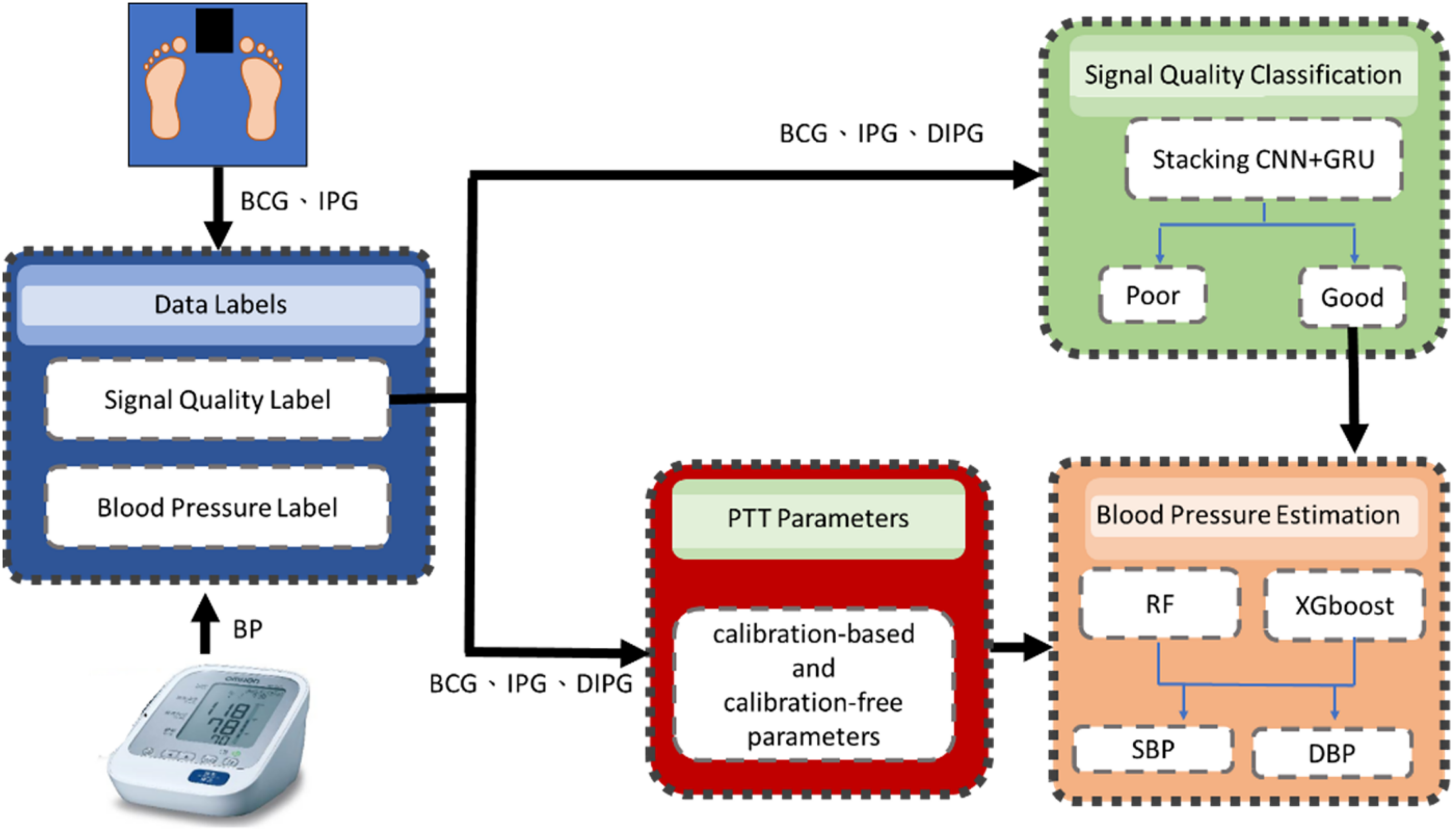
In the flowchart of the proposed method, the blue block is the data labels, the green block is the signal quality classification by a stacking 1D CNN + GRU model, red block is the extraction of calibration-based and calibration-free PTT parameters and the orange block is the BP estimation by RF and XGBoost models.

Figure 1 illustrates the flowchart of the proposed method, comprising four main components: data labeling, signal quality classification, extraction of calibration-based and calibration-free PTT parameters, and BP estimation. BCG and IPG signal are measured from a bodily weight-fat scale, and the reference BP is measured by a digital sphygmomanometer. During the data labeling phase, segment data were annotated with both quality levels and corresponding BP values. Within each segment, PAT parameters were measured from ECG, PPG and DPPG signals as the references, while PTT parameters were measured from BCG, IPG and DIPG signals. The discrepancy between the reference PAT and proposed PTT serves as the criterion for labeling signal quality. The reference BP is measured at one-minute intervals, with BP values between consecutive measurements estimated via linear interpolation. In the signal quality classification stage, a stacked model comprising a 1D CNN and gated recurrent unit (GRU) was employed. This input of the model is the time series data of BCG, IPG, and DIPG. Segments deemed to be of high quality undergo feature engineering. The seven calibration-based and calibration-free PTT parameters are extracted. Subsequently, RF and XGBoost algorithms were separately utilized for BP estimation.

### 2.1 Experiment protocol

Seventeen subjects (11 males and 6 females) were recruited in this study. Their ages, weights, and heights were 20.2 ± 1.1 years (19–22 years) old, 62.8 ± 16.1 kg (43–115 kg), and h166.1 ± 8.0 cm (152–186 cm), respectively. This experiment was approved by the Research Ethics Committee of Chung Shan University Hospital (No. CS2-21194), in Taichung City, Taiwan.

When subjects had rested for about 5 min, they filled out an informed consent form to confirm their participation in this experiment. All subjects had no cardiac disease. Their BPs were measured by a digital sphygmomanometer (HEM-7320, Omron, Osaka, Japan), which served as the reference BP. The cuff was wrapped on the left upper arm. The probe of PPG was placed on the middle finger of the right hand. Two electrodes of ECG were placed on the right and left arms to measure ECG and PPG signals by the self-made circuits (23). A commercial body weight-fat scale (HBF-371, Omron, Osaka, Japan) modified by adding self-made circuits was used to measure the BCG and IPG signals (28). The experiment procedure is described as follows.

1. Subjects stood on the body weight-fat scale for five minutes to measure ECG, PPG, IPG, and BCG signals, with blood pressure measured once.
2. Subjects ran on a treadmill at a fixed speed for at least six minutes to elevate systolic blood pressure (SBP) by 20 mmHg from the resting SBP. When the increase was less than 20 mmHg, subjects were instructed to continue running.
3. After treadmill exercise, subjects stood on the commercial body weight-fat scale for six minutes to measure ECG, PPG, IPG, and BCG signals. Blood pressure was concurrently measured every minute.
4. Each experiment lasted approximately 18 min, and subjects underwent four sessions with an interval of at least one week.

### 2.2 Data processing

**Figure 2:**
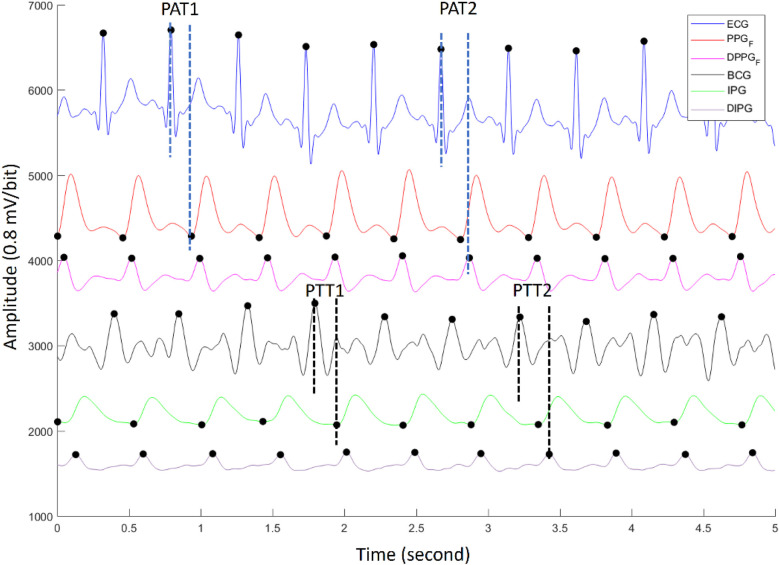
The synchronous ECG (blue), PPG (red), DPPG (pink), BCG (black), IPG (green), and DIPG (purple) signals. The PTT1_BCG_ _+_ _IPG_ and PTT2_BCG_ _+_ _IPG_ are the interval between the J wave of BCG and the foot point of IPG, and the peak point of DIPG. PAT1_ECG_ _+_ _PPG_ and PAT2_ECG_ _+_ _PPG_ are the interval between the R wave of ECG and the foot point of PPG, and the peak point of DPPG.

**Figure 3:**
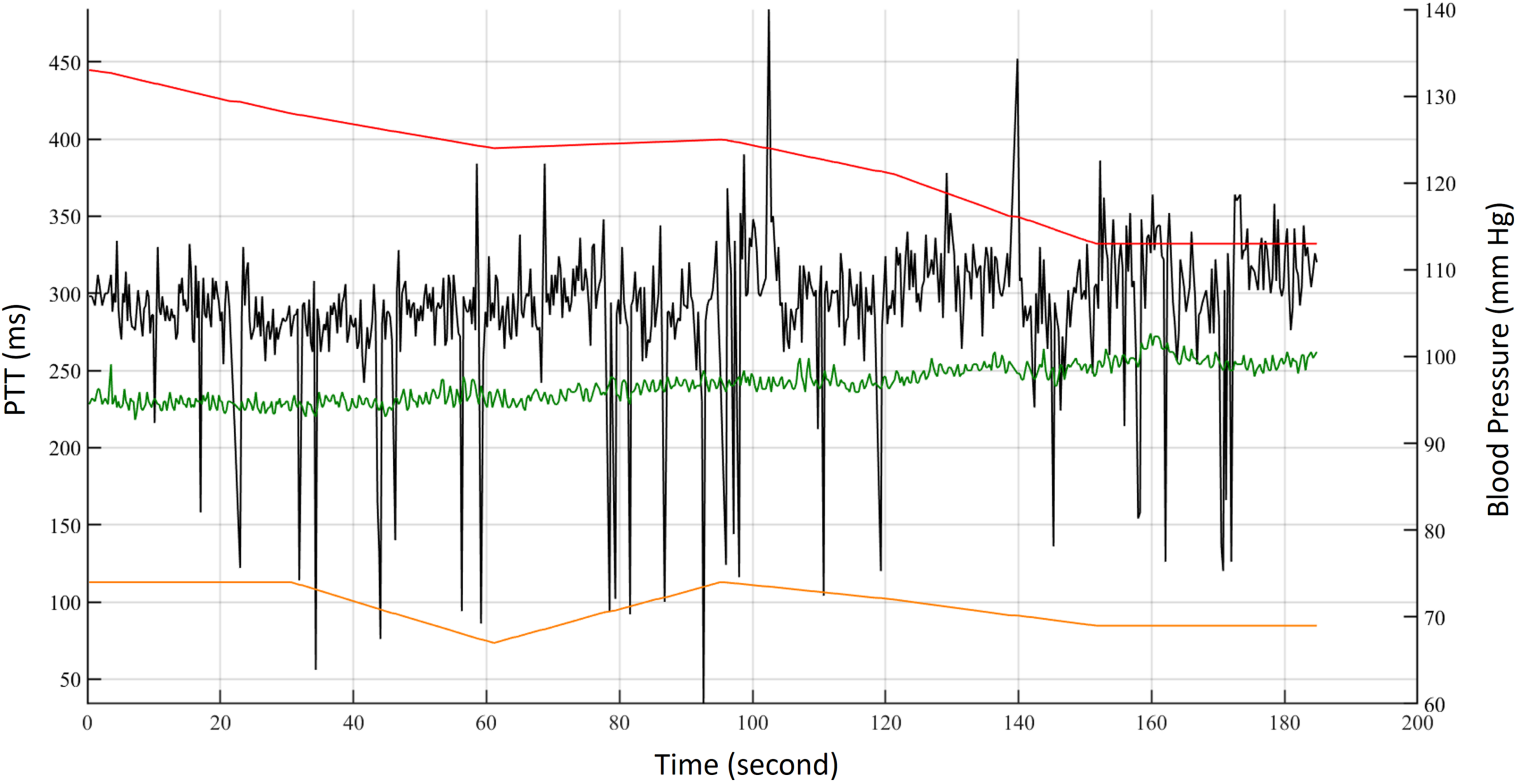
The trends of PAT2_ECG_ _+_ _PPG_ (green), PTT2_BCG_ _+_ _IPG_ (black), SBP (red), and DBP (orange) for one measurement of subject 1.

The ECG, PPG, BCG, and IPG were measured by self-made system with a sampling rate of 500 Hz (28). Then, all measured signals were filtered to remove baseline wandering and high-frequency noise with the 4th-order Butterworth bandpass filter with 0.5–10 Hz 3 dB frequency bandwidth and passed an 8th-order all-pass filter for equalizing the group delay within the passband. Figure 2 shows synchronous ECG (blue), PPG (red), DPPG (pink), BCG (black), IPG (green), and DIPG (purple) signals. The PTT1_BCG_ _+_ _IPG_ is the interval between the J wave of BCG and the foot point of IPG, and the PTT2_BCG_ _+_ _IPG_ is the interval between the J wave of BCG and the peak point of DIPG, as shown in Figure 2. PAT1_ECG_ _+_ _PPG_ is the interval between the R wave of ECG and the foot point of PPG, and PAT2_ECG_ _+_ _PPG_ is the interval between the R wave of ECG and the peak point of DPPG. The R waves of ECG were detected by the Pan-Tompkins method (45). The first zero-crossing points of DIPG and DPPG after an R wave were defined as the foot-point time of IPG and PPG. The J wave of BCG was the first peak after an R wave. Then the first peaks of DIPG and DPPG were detected following their first zero-crossing points. Figure 3 shows the trends of PAT2_ECG_ _+_ _PPG_ (green), PTT2_BCG_ _+_ _IPG_ (black), SBP (red), and DBP (orange) for one measurement of subject 1. The SBP and DBP decrease from 139 mm Hg and 74 mm Hg to 113 mm Hg and 69 mmHg, respectively. The PTT2_BCG_ _+_ _IPG_ and PAT2_ECG_ _+_ _PPG_ increase from 292 ms and 228 ms to 330 ms and 262 ms, respectively. Moreover, the variation of PTT2_BCG_ _+_ _IPG_ is larger than PAT2_ECG_ _+_ _PPG_. In next sector, we used the percentage error between PAT2_ECG_ _+_ _PPG_ and PTT2_BCG_ _+_ _IPG_ to determine the quality of each pulse. Then, the PTT parameters of pulses with the good quality would be used to estimate the BP.

### 2.3 Labeling signal quality and blood pressure

**Figure 4:**
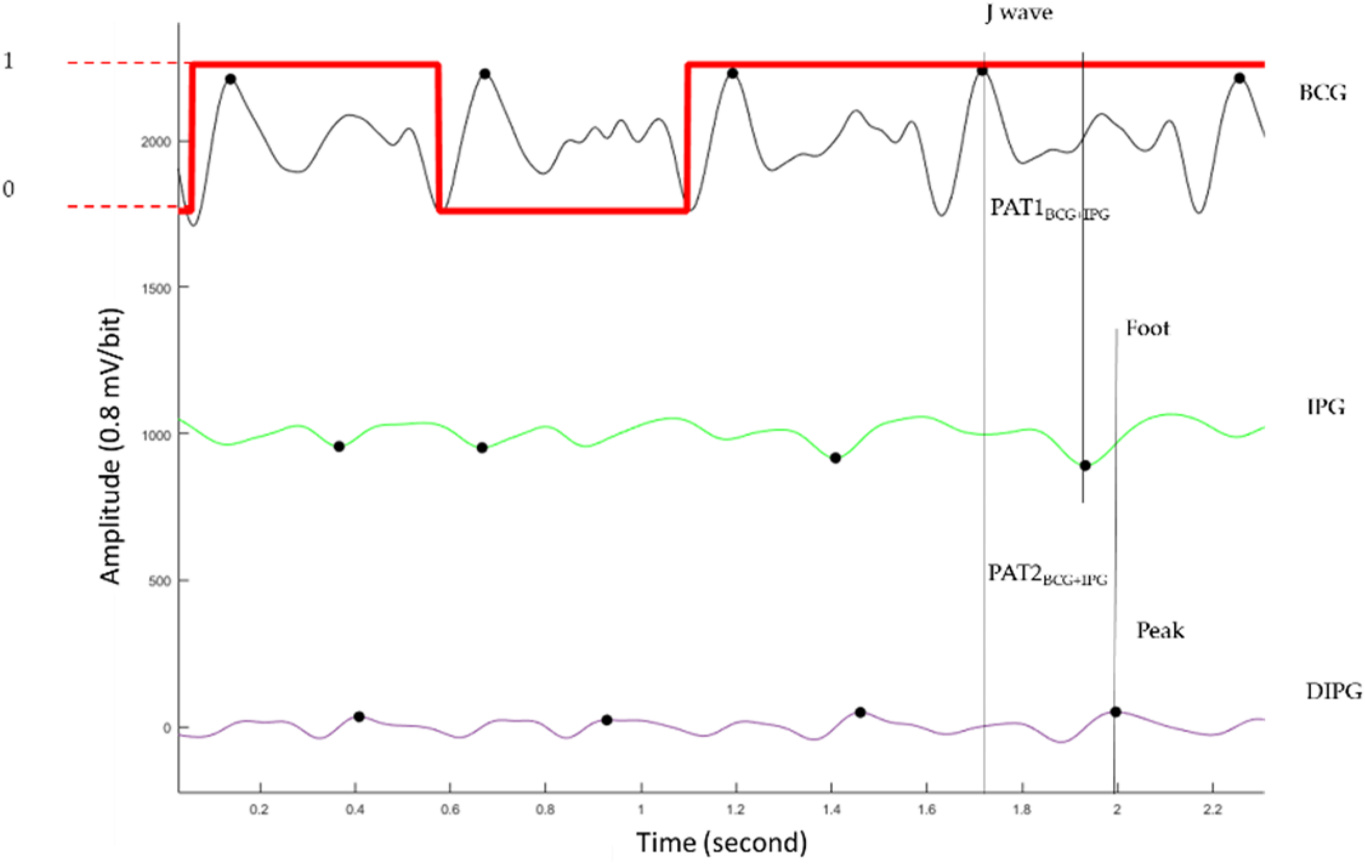
The BCG (black), IPG (green), and DIPG (purple). The label of signal quality for each pulse is the red line. When the pulse belongs to the good quality, the label is 1 (higher horizontal line). Otherwise, the label is 0 (lower horizontal line).

Equation 1 is the percentage error (E) to define the signal quality for each beat, which is the difference between the reference PAT2_ECG_ _+_ _PPG_ and proposed PTT2_BCG_ _+_ _IPG_, and is normalized by the reference PAT2_ECG_ _+_ _PPG_. According to the study of Liu et al. (28), the ECG and BCG have a delay time, as well as PPG and IPG. Thus, the percentage error is defined as,

(1) $$E=\frac{PAT2_{ECG+PPG}-PTT2_{BCG+IPG}+Bias}{PAT2_{ECG+PPG}}\times 100\text{\%}$$

where *Bias* is the average of five delay times between PAT2_ECG_ _+_ _PPG_ and PTT2_BCG_ _+_ _IPG_ measured during rest in each experiment. By the trial and error method, 30% was selected as the threshold. If the *E* is larger than 30%, this pulse is labeled as poor quality. Figure 4 shows the labels of pulses in red. When the pulse is of good quality, the label is 1. Otherwise, the label is 0. The second pulse is labeled as poor quality because the foot of its IPG is in the wrong place.

During the experiment, the subjects’ BP was measured by the digital sphygmomanometer every minute, serving as the reference. We hypothesized that the subjects’ BP tended to decrease after exercise while standing on the weight-fat scale. Thus, we employed the linear interpolation method to estimate the BP for each pulse.

### 2.4 Data segment

**Figure 5:**
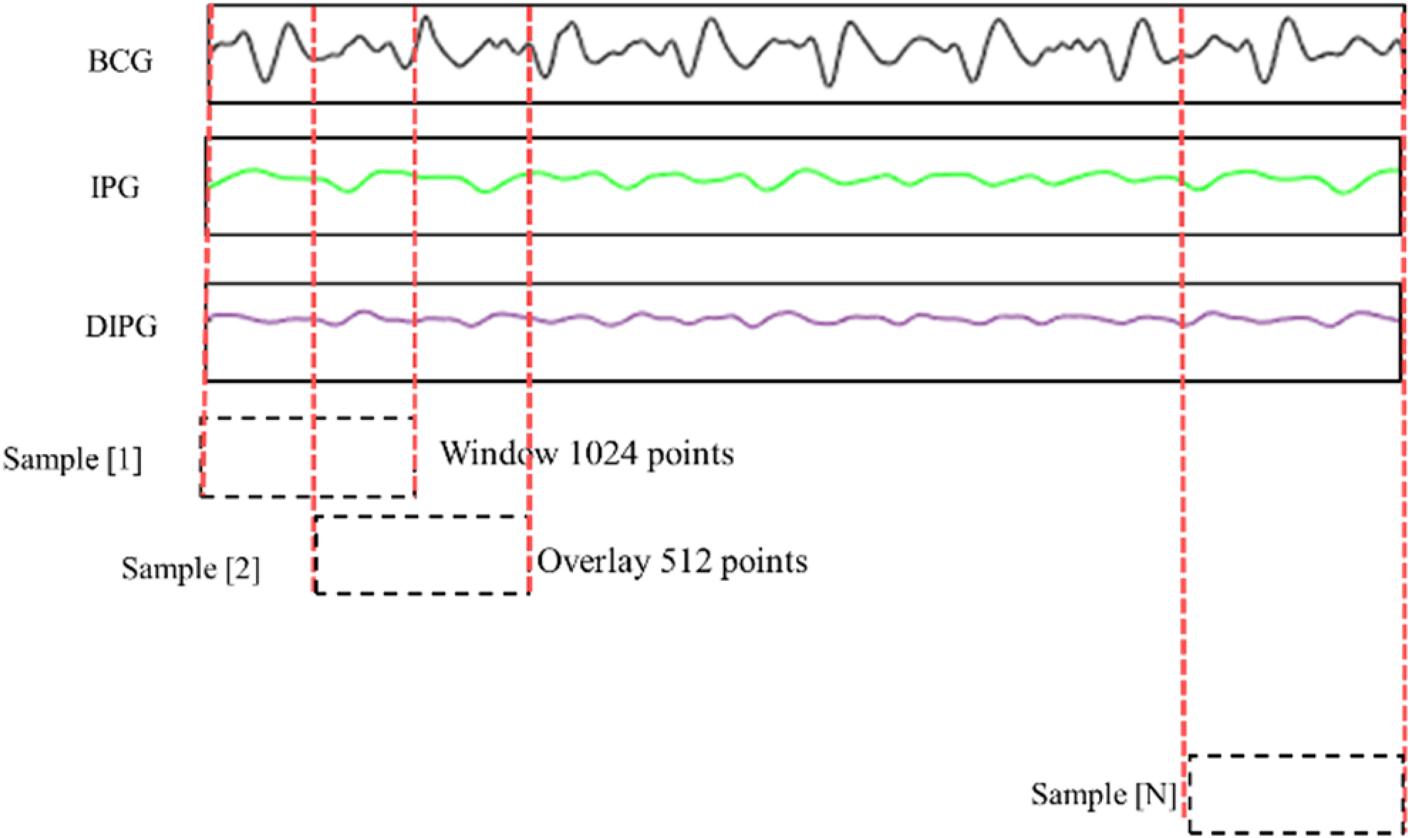
BCG, IPG, and dIPG signals are segmented with 1024 points as a sample, and the overlap is 512 points.

Figure 5 shows the method of data segmentation. The window is of 1024 points with an overlap of 512 points. The segments were labeled as good or poor quality depending on all pulses belonging to good or poor quality. If the quality of one pulse is different from the other pulses, this segment is deleted. The BPs of segments were the average BP of all pulses. The time of a segment is about 2 s, and the overlapping time is about 1 s. Thus, the numbers of good and poor quality samples were 2,262 and 20,358, a total of 22,620 samples. The BCG, IPG, and DIPG signals in the segment were normalized by Equation (2).

(2) $$Z=\frac{x-\bar{x}}{s}$$

where Z is the normalized signal, *x* is the original signal, and its mean and standard deviation are $\bar{x}$ and *s*, respectively.

The samples with good quality were used to estimate the BP. There were 2262 samples. According to the IEEE Standard for Wearable, Cuffless Blood Pressure Measuring Devices (46), the BP classification has four categories, normal (SBP < 120 mmHg and DBP < 80 mmHg), pre-hypertension (120 mmHg ≤ SBP < 140 mmHg or 80 mmHg ≤ DBP < 90 mmHg), stage-1 hypertension (140 mmHg ≤ SBP < 160 mmHg or 90 mmHg ≤ DBP < 100 mmHg), and stage-2 hypertension (160 mmHg ≤ SBP or 100 mmHg ≤ DBP). In the 2,262 samples, the numbers of four BP categories are 597, 766, 785, and 114, respectively.

### 2.5 Model of signal quality classification

**Figure 6:**
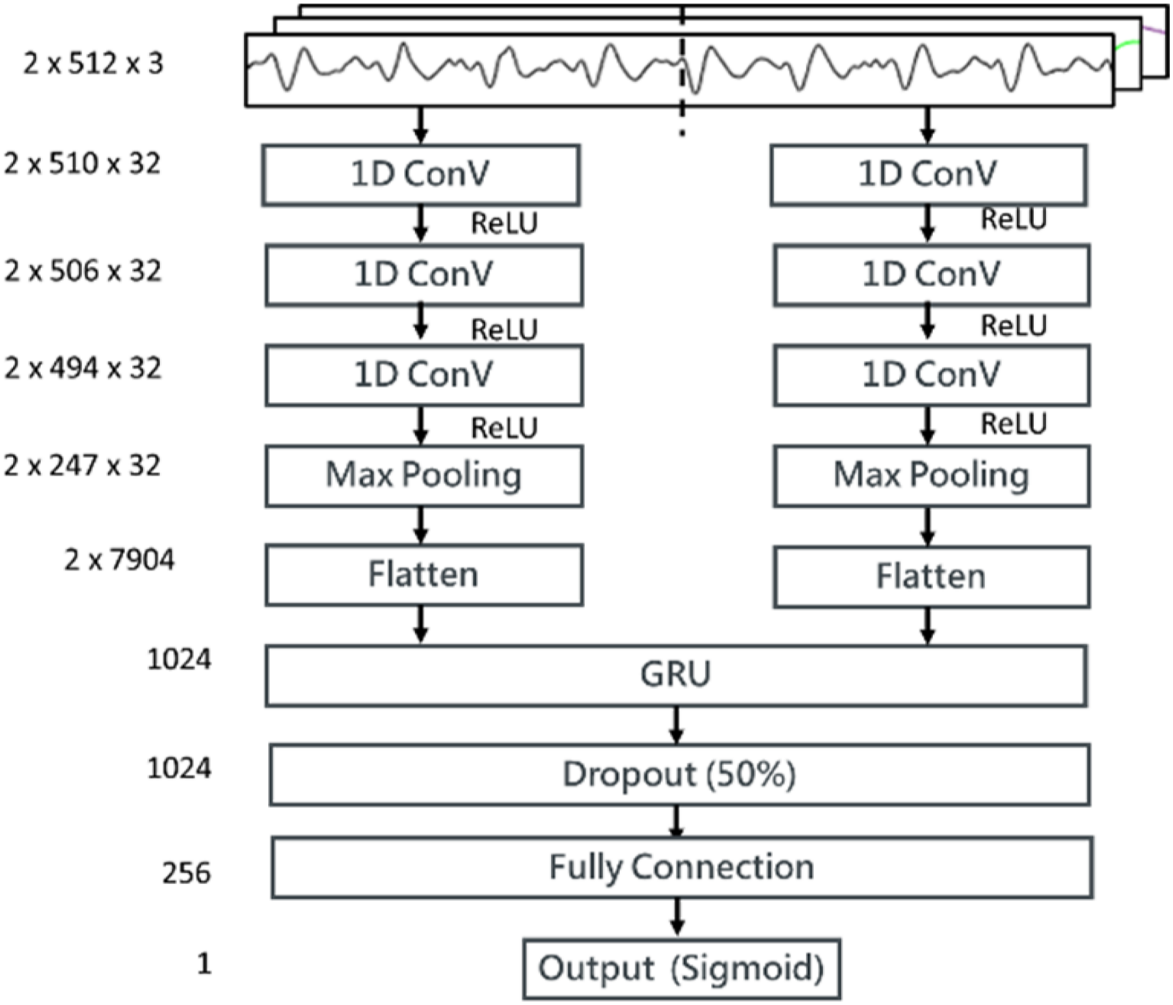
The structure of the proposed stacking 1D CNN + GRU model for the signal quality classification. Three input channels, BCG, IPG, and DIPG come through a time-distributed layer comprising a stacking 1D CNN + GRU. Then, weights between GRU layer and full connection layer are dropout 50%. The activation function in output layer is sigmoid function.

We proposed a stacked 1D CNN + GRU model for the signal quality classification as shown in Figure 6. The input sample was a time series data of three channels, BCG, IPG, and DIPG. A time-distributed layer comprising two 1D CNNs (i.e., two pairs of CNNs with three layers, a maximal pool layer, and a flattened layer) is stacked on the top of GRU. A sample was separated into two segments, each containing 512 points. In the convolutional layer, the number of filters is 32, the kernel sizes are 3, 5, and 13, respectively, and the stride is 2. In the maximal pooling layer, the kernel size is 2, and the stride is 2. The activation function employed is ReLU. The unit number of GRU is set to 1,024. The batch size is 512, with the control reset gate and update gate using a sigmoid function and the hidden state using a tanh function. The numbers of full connection layers are 1024, 256, and 1, respectively, with ReLU as the activation function in convolutional layers and hidden layers and the sigmoid function in the output layer. The threshold of output is 0.5. The dropout in the hidden layer is 0.5. The loss function is the binary Cross-Entropy function, and the Adam optimizer is used, with a learning rate of 0.0001.

### 2.6 Parameters of blood pressure estimation

**Figure 7:**
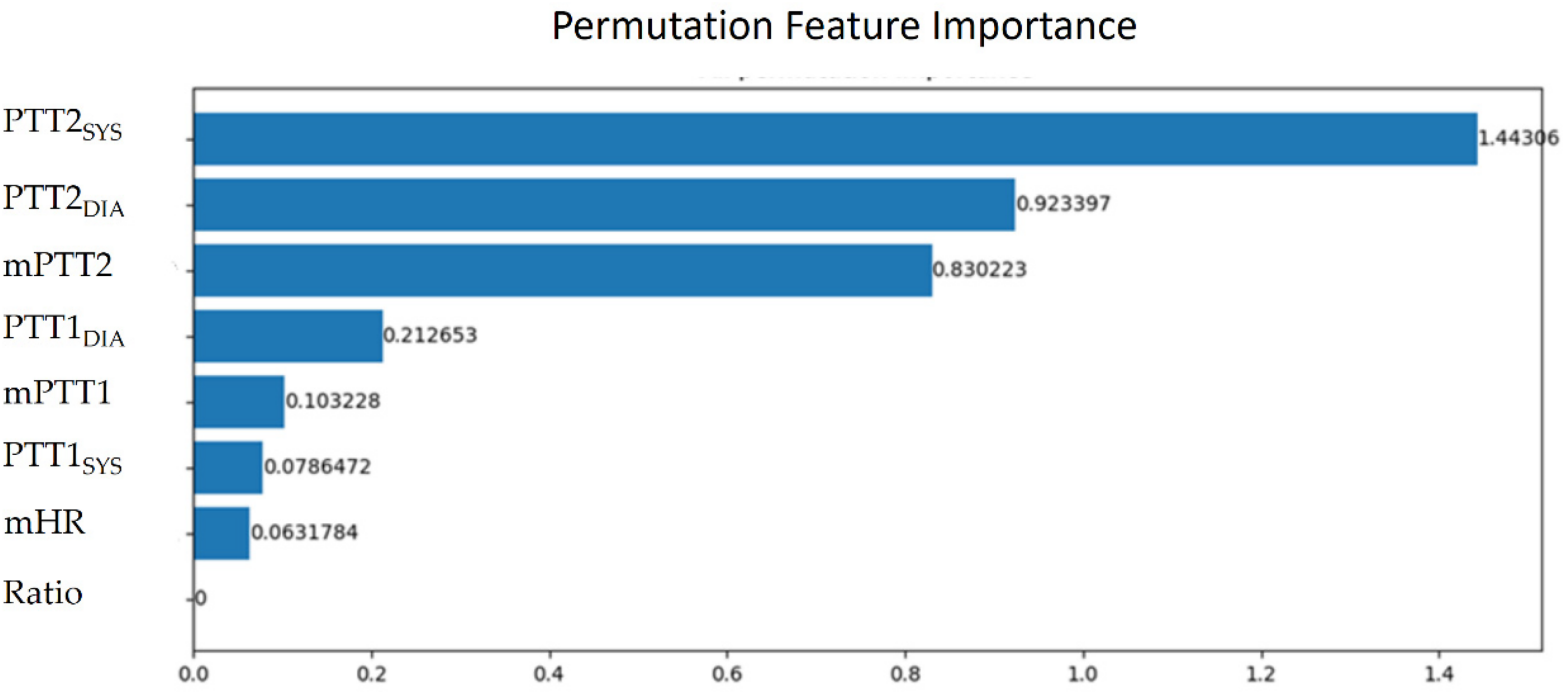
The significant scores of mPTT2, PTT2_SYS_, PTT2_DIA_, mPTT1, PTT1_SYS_, PTT1_DIA_, mHR, and ratio with the permutation feature importance.

The parameters of BP estimation were extracted from the pulses with good quality. Because the size of samples was 1024 points, there were at least two pulses in a sample. Thus, a sample has at last two PTT parameters. In a sample, mPTT1, mPTT2, and mHR denote the averages of all PTT1_BCG_ _+_ _IPG_ values, all PTT2_BCG_ _+_ _IPG_ values, and all heart rates, respectively. SBP_rest_ and DBP_rest_ are the BP in the resting phase. Table 1 shows the definition of eight parameters in a sample. The calibration-based PTT parameters are the ratios between PTT parameters and SBP_rest_ and DBP_rest_, respectively, including PTT1_SYS_, PTT1_DIA_, PTT2_SYS_, and PTT2_DIA_. The calibration-free PTT parameters include mPTT1, mPTT2, and Ratio. Table 1 shows the definition of eight parameters. Then, we evaluated the important degrees of eight parameters. Permutation feature importance is a model inspection technique, and a useful method for nonlinear estimation (47). When a single parameter is shuffled randomly, the model score is decreasing. This drop represents how much the model depends on this parameter. This procedure will break the relation between the parameter and the target. This method will be calculated many times with the different permutations of parameters. The higher score, the higher significance. Figure 7 shows the significant scores of eight parameters with this method based on XGBoost. The scores of mPTT2, PTT2_SYS_, PTT2_DIA_, mPTT1, PTT1_SYS_, PTT1_DIA_, HR, and Ratio are 1.44, 0.92, 0.83, 0.21, 0.10, 0.08, 0.06, and 0, respectively. Since the score for Ratio is 0, the other seven parameters, excluding Ratio, were used to explore the model with the best performance.

**Table 1:** The definition of parameters in a sample.

Parameter	Formula	Description
mPTT1		Average of PTT1
mPTT2		Average of PTT2
Ratio	$\frac{mPTT1}{mPTT2}$	Ratio between mPTT1 and mPTT2
PTT1_SYS_	$\frac{mPTT1}{SBP_{rest}}$	Ratio between mPTT1 and SBP_rest_
PTT1_DIA_	$\frac{mPTT1}{DBP_{rest}}$	Ratio between mPTT1 and DBP_rest_
PTT2_SYS_	$\frac{mPTT2}{SBP_{rest}}$	Ratio between mPTT2 and SBP_rest_
PTT2_DIA_	$\frac{mPTT2}{DBP_{rest}}$	Ratio between mPTT2 and DBP_rest_
mHR		Average of heart rates

### 2.7 Regression models of blood pressure

This study employed two prevalent nonlinear regression models, RF and XGBoost, both developed by Python.

#### 2.7.1 Random forest

The RF model represents an advanced extension of the decision tree model, wherein random trees are constructed across multiple subspaces to reduce inter-correlation among them (40). For this study, the minimum number of leaves, the minimum number of splits, and the number of trees were set to 4, 2, and 300, respectively.

#### 2.7.2 XGBoost

XGBoost (eXtreme Gradient Boosting) represents an enhanced version of the Gradient Boosting technique, combining numerous weak decision trees to construct a powerful predictive model (48). Compared to conventional classification and regression techniques, XGBoost typically demonstrates a superior accuracy due to its robustness and adaptive learning capability. For this study, the learning rate, maximum depth, and the number of trees were set to 0.1, 3, and 300, respectively.

### 2.8 Statistical analysis

The quantitative data is expressed as the mean ± standard deviation. In the classification of signal quality, the sensitivity, specificity, and accuracy are used to evaluate the performance of model.

(3) $$Sensitivity(\text{\%})=\frac{TP}{\left(TP+FN\right)}\times 100\text{\%}$$

(4) $$Specificity(\text{\%})=\frac{FP}{\left(FP+TN\right)}\times 100\text{\%}$$

(5) $$Accuracy(\text{\%})=\frac{TP+TN}{\left(TP+TN+FP+FN\right)}\times 100\text{\%}$$

TP, FN, FP, and TN are true positive, false negative, false positive, and true negative, respectively.

Moreover, the Pearson correlation coefficient (PCC), *ρ*, is used to analyze the relationship between the estimated and target BPs. Equation (6) shows the calculation of correlation coefficient,

(6) $$\rho (X,Y)=\frac{\sum_{i=1}^{n}\;(x_{i}-\bar{x})(y_{i}-\bar{y})}{\sqrt{\sum_{i=1}^{n}\;{\left(x_{i}-\bar{x}\right)}^{2}}\sqrt{\sum_{i=1}^{n}\;{\left(y_{i}-\bar{y}\right)}^{2}}}$$

where X and Y are the estimated and target BPs, and *n* is the number of testing samples. The mean absolute difference (MAD) is also used to confirm the accuracy of the proposed model,

(7) $$MAD=\frac{S^{2}+{\bar{D}}^{2}}{\sqrt{2S^{2}+{\bar{D}}^{2}}},$$

where S is the standard deviation of errors, and ${\bar{D}}^{2}$ is the mean of error. Furthermore, the precision and agreement between the standard and estimated BP by the reference and proposed methods are compared using a Bland–Altman plot.

## 3 Results

The hardware utilized in this study consisted of an Intel Core i7-8700 CPU and an NVIDIA GeForce GTX3070 GPU. The results include two parts, signal quality classification and BP estimation. In the signal quality classification, there were 22,620 samples, with 15,834 and 6,786 samples allocated for training and testing, respectively. For BP estimation, only samples with good quality were used, resulting in 2,262 samples, with 1,809 and 453 samples for training and testing, respectively. The training samples were divided into training and validation sets in an 8:2 ratio.

### 3.1 Signal quality classification

**Figure 8:**
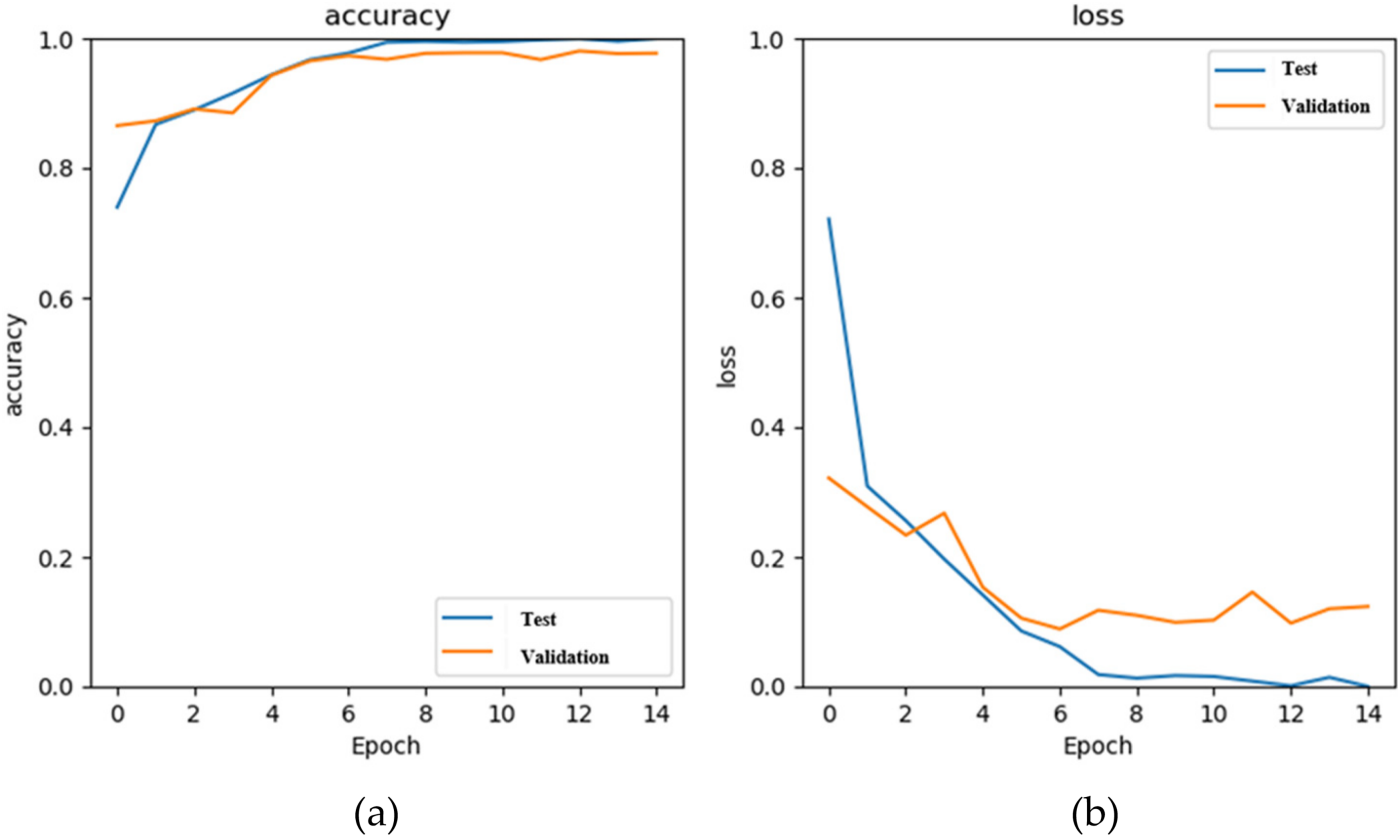
The results of the stacking 1D CNN + GRU model in the training (blue) and validation (orange) phases, **(a)** the accuracy curves, and **(b)** the loss curves.

**Figure 9:**
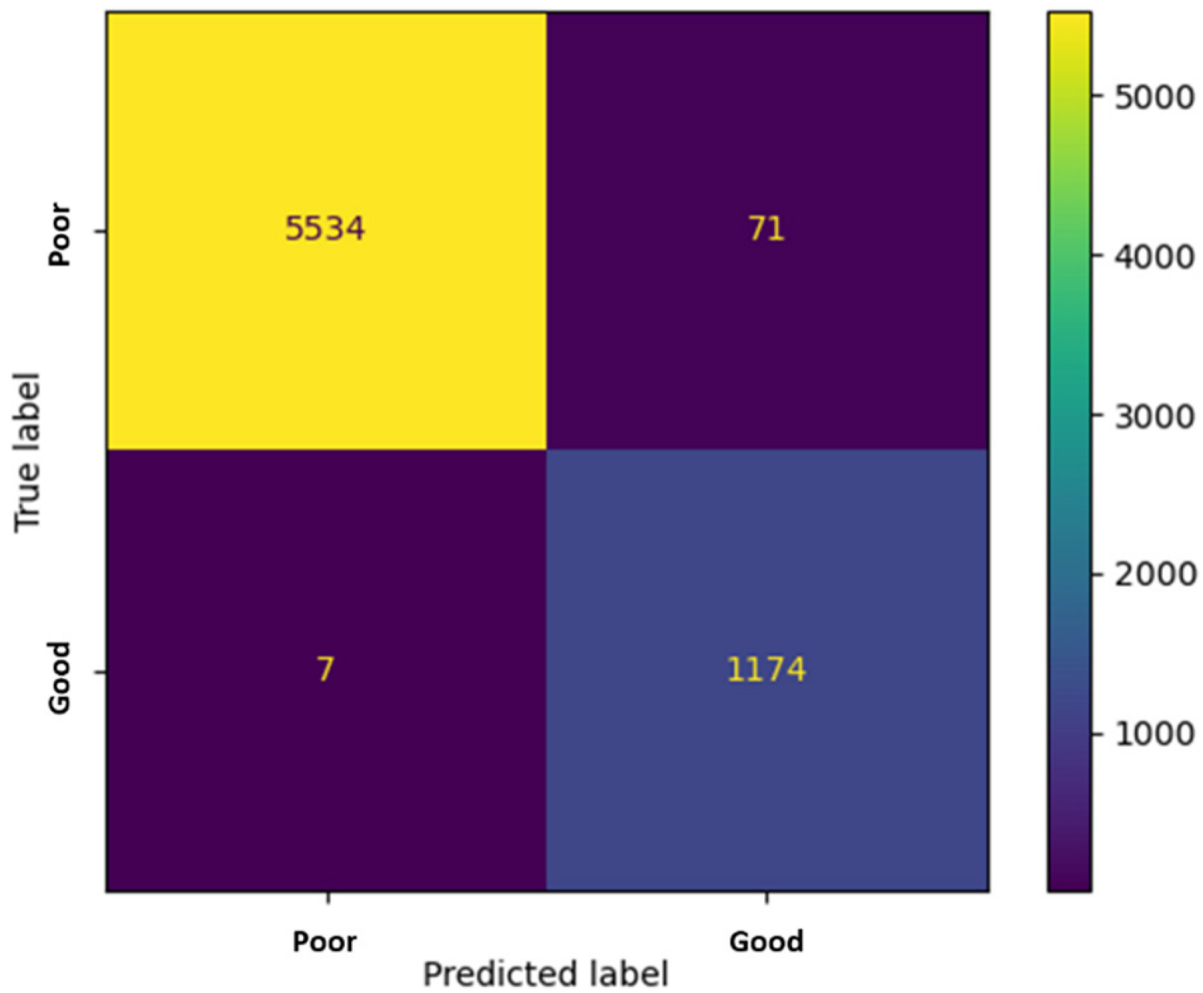
The fusion matrix in the testing phase. The numbers of TP, TN, FN, FP are 1174, 5534, 7, and 71, respectively.

Figure 8a shows the accuracy curves of the stacking 1D CNN + GRU model in the training and validation phases, and Figure 8b illustrates the loss curves. The blue and orange lines are the training and validation results of each epoch. When the number of epochs is 14, the accuracy approaches 0.97, and the loss value approaches 0.12. Figure 9 shows the fusion matrix, the numbers of TP, TN, FN, FP are 1174, 5534, 7, and 71, respectively. The accuracy, sensitivity, and specificity are 0.989, 0.994, and 0.987, respectively.

### 3.2 Blood pressure estimation

**Figure 10:**
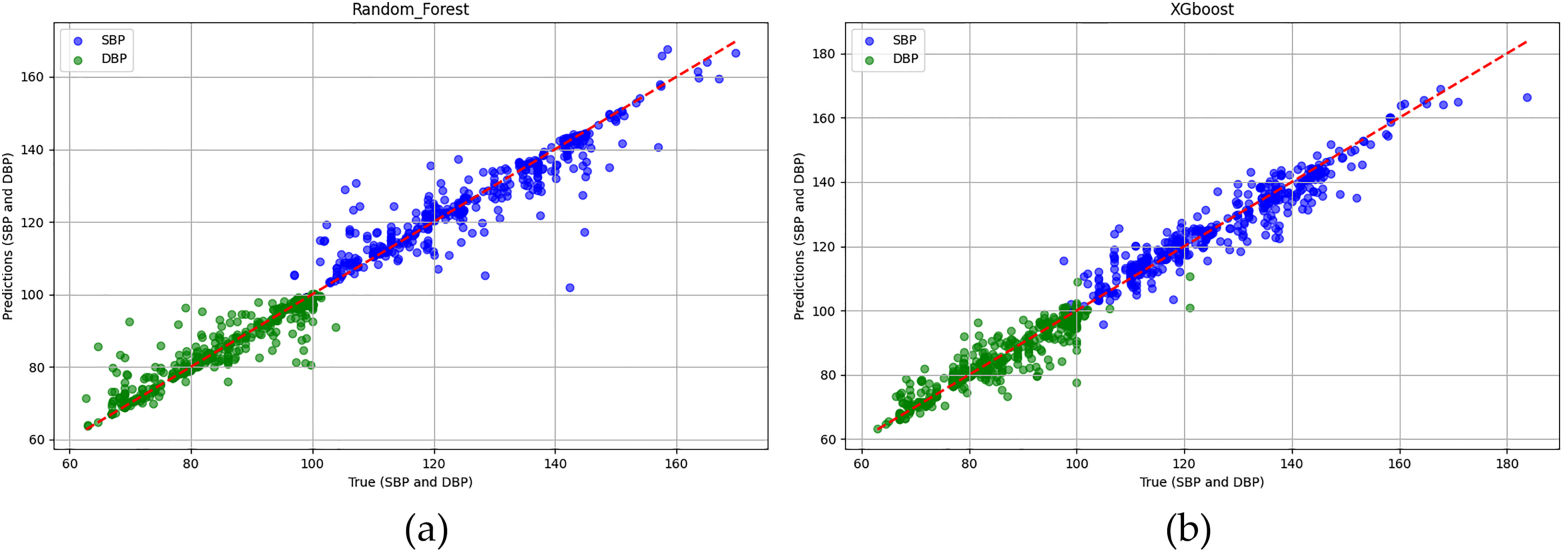
The Pearson correlation coefficients of SBP (blue circle) and DBP (green circle) estimations **(a)** RF model are both 0.94. **(b)** XGBoost model are 0.95 and 0.94, respectively.

**Figure 11:**
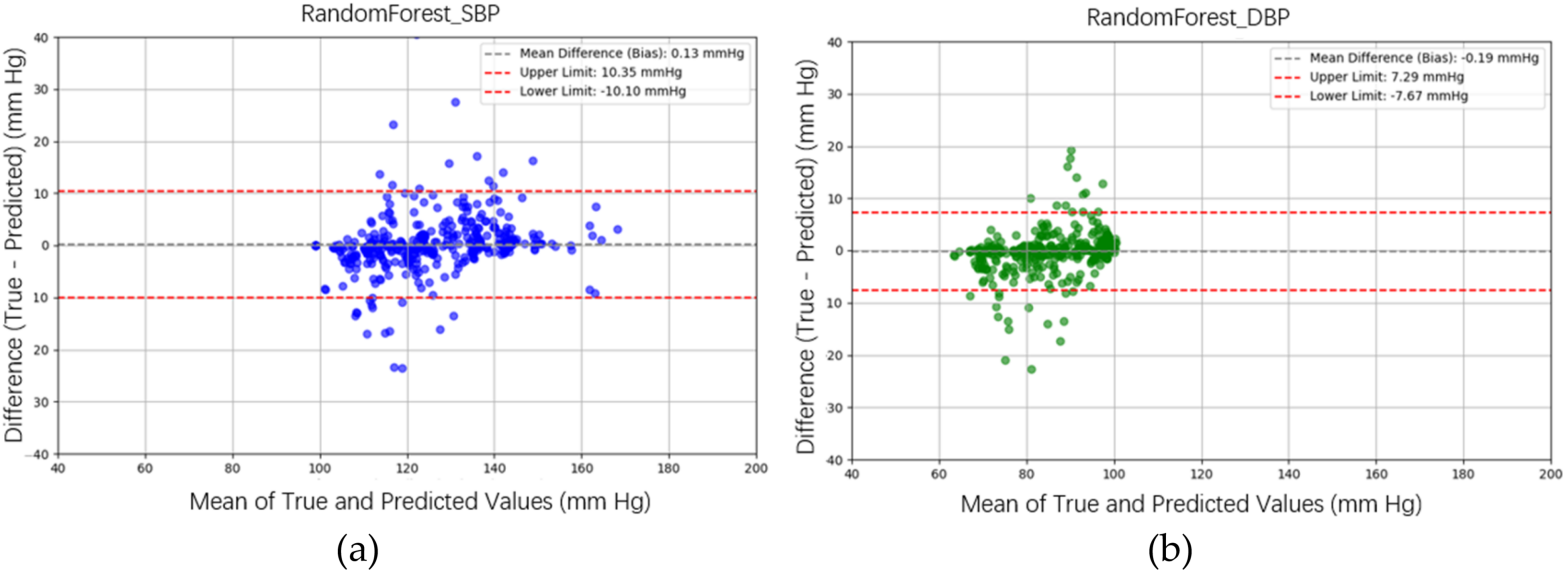
Bland–Altman plots for SBP and DBP estimations using the RF model are depicted. **(a)** For SBP estimation, a bias of 0.13 mmHg is observed, with the upper and lower bounds of agreement at 10.35 and −10.10 mmHg, respectively. **(b)** For DBP estimation, a bias of −0.19 mmHg is noted, with the upper and lower bounds of agreement at 7.29 and −7.67 mmHg, respectively.

**Figure 12:**
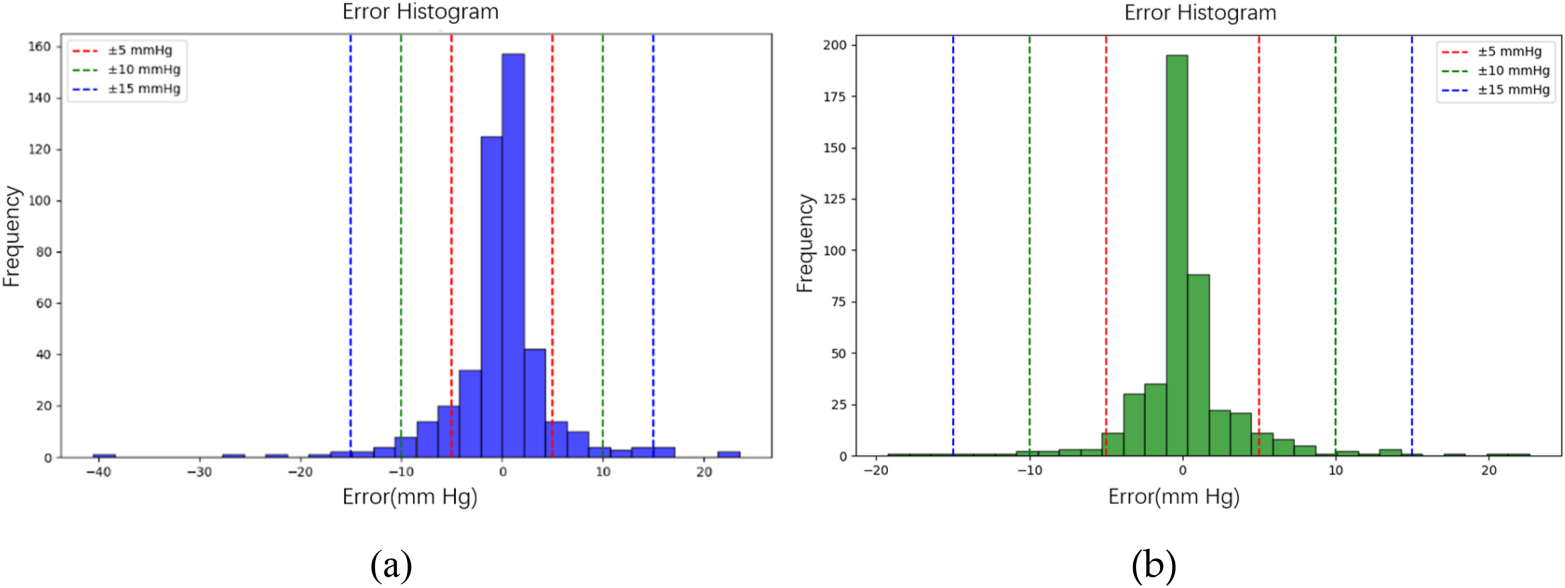
The distributions of absolute difference between reference and BP estimation by RF model. **(a)** SBP estimated with 83.2%, 93.8%, and 97.4% differences no more than 5 mmHg, 10 mmHg, and 15 mmHg, respectively. **(b)** DBP estimated with 89.4%, 96.0%, and 98.5% differences less than 5 mmHg, 10 mmHg, and 15 mmHg, respectively.

In the testing phase, the numbers of samples in the categories of normal, pre-hypertension, stage-1 hypertension, and stage-2 hypertension were 122, 146, 155, and 30, respectively. Figure 10a illustrates the BP estimation using the RF model, with PCCs of 0.94 for both SBP (blue circle) and DBP (green circle). The MAD were 3.54 mmHg for SBP and 2.57 mmHg for DBP. Bland–Altman plots for SBP are depicted in Figure 11a, indicating a bias of 0.13 mmHg, with upper and lower bounds of agreement at 10.35 and −10.10 mmHg, respectively. Similarly, Bland–Altman plots for DBP, shown in Figure 11b, display a bias of −0.19 mmHg, with upper and lower bounds of agreement at 7.29 and −7.67 mmHg, respectively. The percentages of samples falling outside the limits of agreement for SBP and DBP were 5.74% and 6.18%, respectively. Figure 12 shows the distributions of absolute difference between reference and BP estimation. SBP estimated by RF, in Figure 12a, are 83.2%, 93.8%, and 97.4% differences no more than 5 mmHg, 10 mmHg, and 15 mmHg, respectively. DBP estimated by RF, in Figure 12b, are 89.4%, 96.0%, and 98.5% differences less than 5 mmHg, 10 mmHg, and 15 mmHg, respectively.

**Figure 13:**
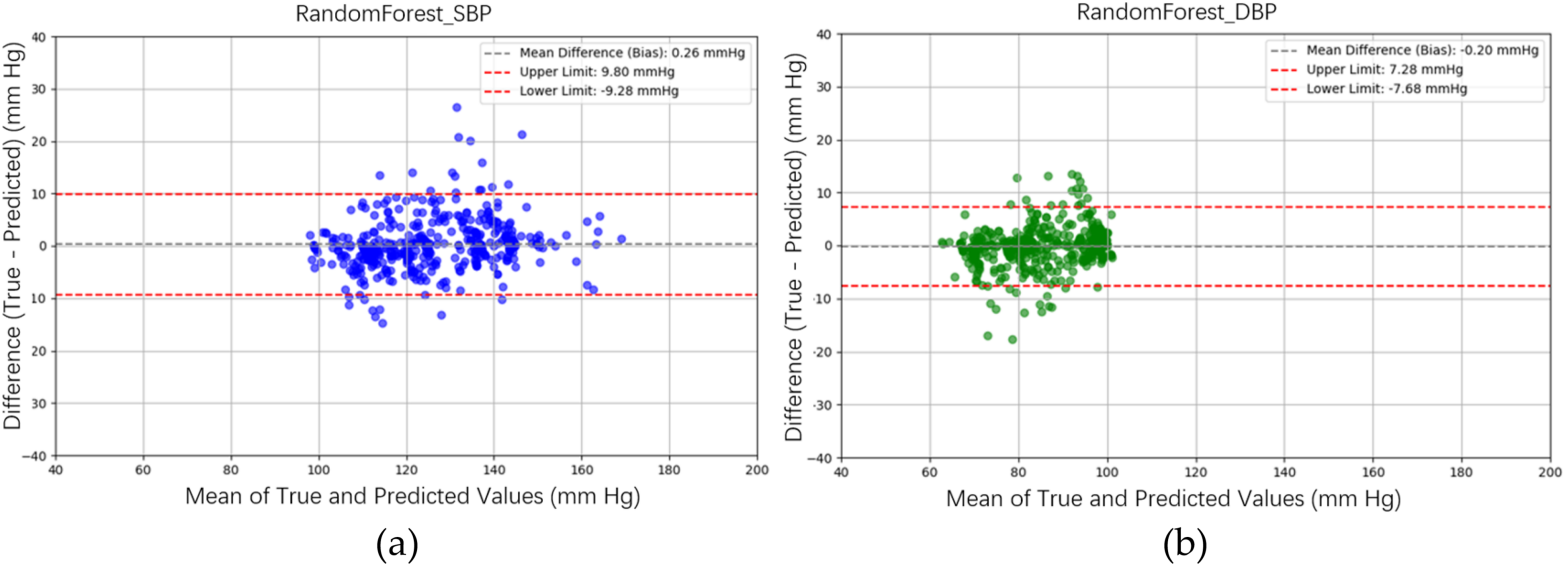
Bland–Altman plots for SBP and DBP estimations using the XGBoost model are depicted. **(a)** For SBP estimation, a bias of 0.26 mmHg is observed, with the upper and lower bounds of agreement at 9.80 and −9.28 mmHg, respectively. **(b)** For DBP estimation, a bias of −0.20 mmHg is noted, with the upper and lower bounds of agreement at 7.28 and −7.68 mmHg, respectively.

**Figure 14:**
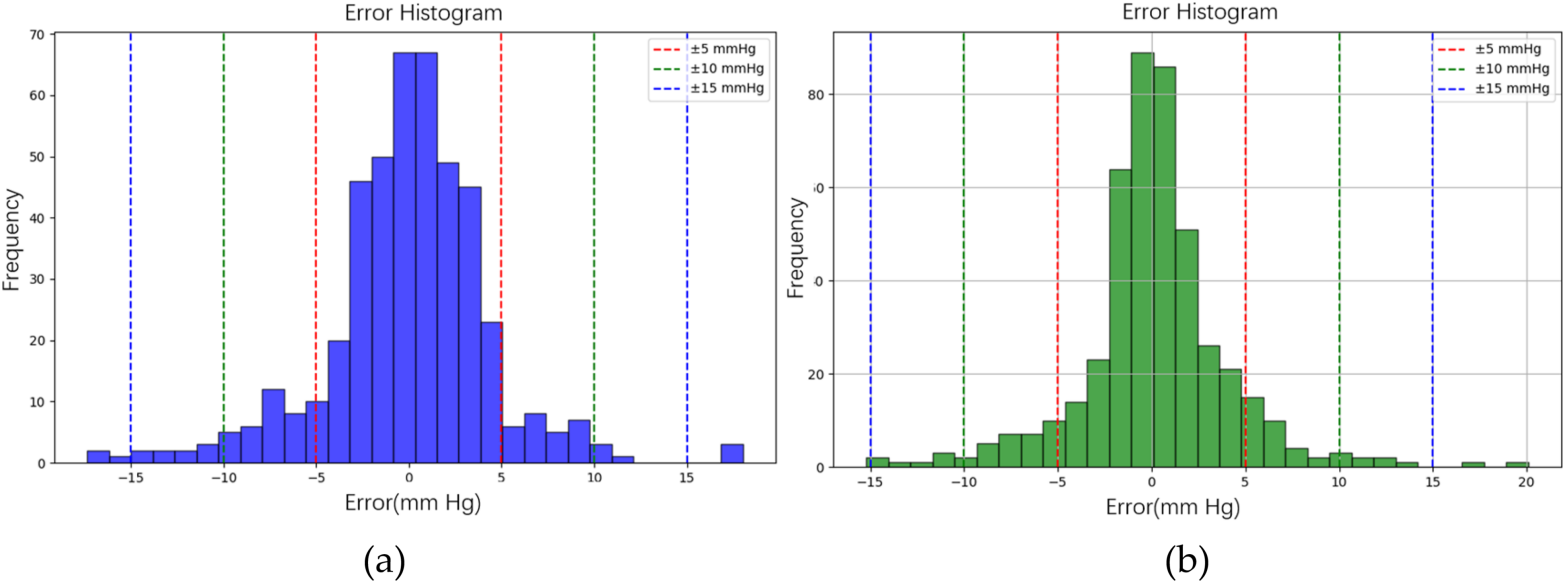
The distributions of absolute difference between reference and BP estimation by XGBoost model. **(a)** SBP estimated with 81.9%, 95.6%, and 98.7% differences no more than 5 mmHg, 10 mmHg, and 15 mmHg, respectively. **(b)** DBP estimated with 83.9%, 96.9%, and 99.1% differences less than 5 mmHg, 10 mmHg, and 15 mmHg, respectively.

**Figure 15:**
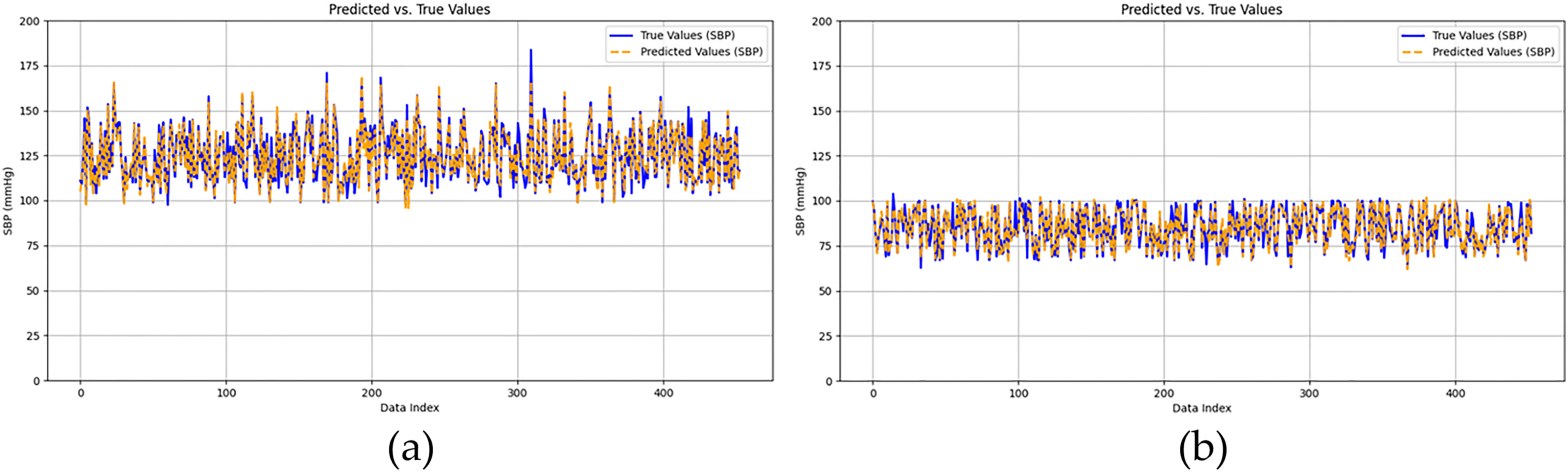
The illustrations of the reference BP and BP estimated by xGboost, **(a)** SBP, **(b)** DBP.

Figure 10b illustrates the BP estimation using the XGBoost model, with Pearson correlation coefficients (PCCs) of 0.95 for SBP (blue circle) and 0.94 for DBP (green circle). The MAD for SBP and DBP were 3.35 and 2.67 mmHg, respectively. Bland–Altman plots for SBP are presented in Figure 13a, indicating a bias of 0.26 mmHg, with upper and lower bounds of agreement at 9.80 and −9.28 mmHg, respectively. Similarly, Bland–Altman plots for DBP, shown in Figure 13b, exhibit a bias of −0.20 mmHg, with upper and lower bounds of agreement at 7.28 and −7.68 mmHg, respectively. The percentages of samples falling outside the limits of agreement for SBP and DBP were 5.74% and 6.62%, respectively. Figure 14 shows the distributions of absolute difference between reference and BP estimation by XGBoost. SBP estimation, in Figure 14a, are 81.9%, 95.6%, and 98.7% differences no more than 5 mmHg, 10 mmHg, and 15 mmHg, respectively. DBP estimation, in Figure 14b, are 83.9%, 96.9%, and 99.1% differences less than 5 mmHg, 10 mmHg, and 15 mmHg, respectively. Figure 15. Shows the illustrations of the reference BP and BP estimated by XGboost with testing samples, (a) SBP, (b) DBP.

For the 5-fold cross-validation results of the RF and XGBoost models, in Table 2, PCCs for SBP estimation are 0.947 ± 0.010 and 0.953 ± 0.007, respectively. PCCs in DBP estimation are 0.940 ± 0.007 and 0.935 ± 0.007 for RF and XGBoost models, respectively. In Table 3, the MADs of SBP estimation for RF and XGBoost models are 3.54 ± 0.34 and 3.35 ± 0.28 mmHg, respectively. Moreover, the MADs of DBP estimation for RF and XGBoost models are 2.57 ± 0.17 and 2.67 ± 0.15 mmHg, respectively. The training and testing times for a single sample in the RF model are 758 µs and 40 µs, respectively, whereas, for the XGBoost model, they are 111 and 7 µs, respectively. The model sizes for the RF and XGBoost models are 5.04 and 0.31 Mbytes, respectively.

**Table 2:** PCCs and MAD of each fold in 5-fold cross-validation.

PCC	First fold	Second fold	Third fold	Fourth fold	Fifth Fold	Total
RF_SBP	0.9381	0.9392	0.9386	0.9592	0.9591	0.947 ± 0.010
RF_DBP	0.9357	0.9363	0.9445	0.9519	0.9322	0.940 ± 0.007
XGBoost_SBP	0.9478	0.9470	0.9468	0.9611	0.9606	0.953 ± 0.007
XGBoost_DBP	0.9367	0.9327	0.9384	0.9440	0.9606	0.935 ± 0.007
MAD						
RF_SBP (mmHg)	3.6861	3.8243	3.8531	3.1792	3.1652	3.54 ± 0.34
RF_DBP (mmHg)	2.7045	2.6671	2.4550	2.3341	2.7136	2.57 ± 0.17
XGBoost_SBP (mmHg)	3.3960	3.5830	3.5974	3.084	3.0863	3.35 ± 0.28
XGBoost_DBP (mmHg)	2.6735	2.7439	2.5802	2.4987	2.8775	2.67 ± 0.15

**Table 3:** The comparison with the previous studies for signal quality assessment of PPG.

Reference	Model	Data	Results
(34)	ResNet-50	2D image	Accuracy: 0.925
(35)	SVM	1D features	Accuracy: 0.899
(36)	RF	1D features	Accuracy: 0.968
(37)	Lightweight CNN	2D image	Accuracy: 0.975
(38)	6-layer CNN	1D signal	Accuracy: 0.978 Specificity: 0.948 Sensitivity: 0.993
Proposed method	Stacked CNN + GRU	1D signals	Accuracy: 0.989 Specificity: 0.987 Sensitivity: 0.994

## 4 Discussion

**Figure 16:**
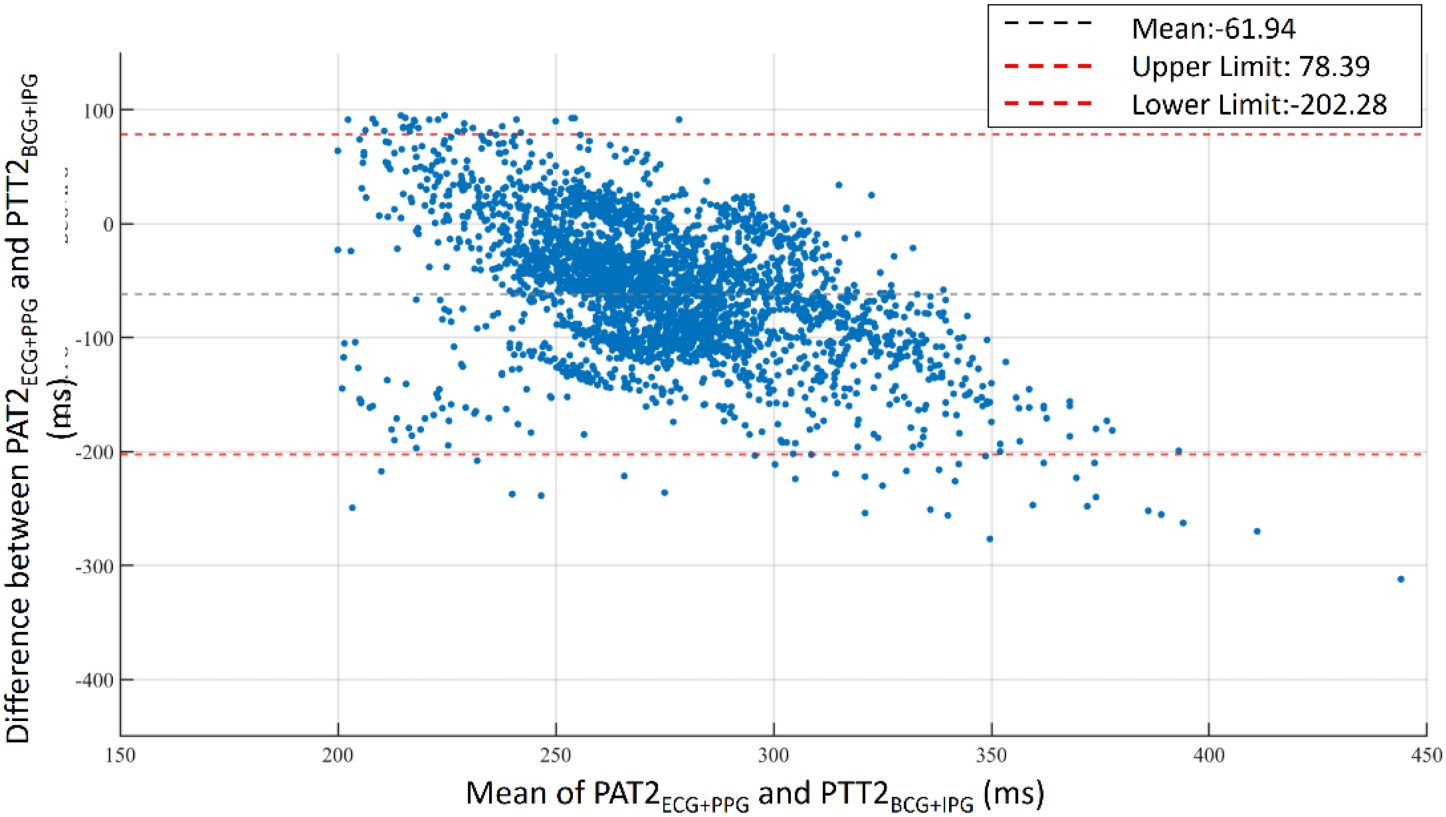
Bland–Altman plot for PAT2_ECG_ _+_ _PPG_ and PTT2_BCG_ _+_ _IPG_. A bias of −61.94 ms is observed with upper and lower bounds of agreement at 78.39 ms and −202.28 ms, respectively.

The bathroom weight-fat scale has become a popular healthcare device, utilizing strain gauges and bioimpedance analysis to measure body weight and body fat ratio (49). BCG and IPG signals are embedded in the weight and body impedance measurements. Inan et al. utilized a weight-fat scale to measure ECG, BCG, and IPG signals using custom-designed circuits, and analyzed the signal-to-noise ratios (SNRs) of these signals (29). They identified the main disadvantage of this method as the interference caused by electromyogram (EMG). To address this, Inan et al. used BCG and seismocardiogram signals measured by an accelerometer placed on the hand (17) to mitigate interference with EMG. Liu et al. suggested that the SNR of IPG could be affected by the permutation of electrodes (27). Therefore, during the measurement of PTT using BCG (proximal reference) and IPG (distal reference), signal quality is a significant challenge. In this study, the reference PAT was measured by ECG and PPG, which was the classical method of cuffless BP measurement (14). In Figure 3, we also find that the variation of PAT2_ECG_ _+_ _PPG_ is lower than PTT2_BCG_ _+_ _IPG_. We defined the percentage error of 30% to tag the label of signal quality. This method could decrease the human subjective influence. Moreover, the lower percentage error, the higher the accuracy of BP measurement. The number of samples would also decrease. Figure 16 shows the Bland–Altman plot for PAT2_ECG_ _+_ _PPG_ and PTT2_BCG_ _+_ _IPG_ to exhibit a bias of −61.94 ms, with upper and lower bounds of agreement at 78.39 ms and −202.28 ms, respectively.

The PTT information is embedded in the time-sequence data of BCG, IPG, and DIPG. In instances where these signals are of poor quality, temporal features exhibit significant variation. The stacked 1D CNN + GRU model includes two parts: feature extraction using 1D CNN layers and temporal sequence recognition using the GRU layer. This model has previously been employed for human activity recognition using inertial sensors (50). In this study, the BCG, IPG, and DIPG signals were directly used to train the proposed model, the stacked 1D CNN + GRU. The accuracy, sensitivity, and specificity approached 0.989, 0.994, and 0.987, respectively. We did not find any study to classify the signal quality of synchronous BCG and IPG signals. Thus, Table 5 shows the previous studies for signal quality assessment of PPG with ML or deep learning techniques. We find that the accuracies of these studies (36–38) are very close. However, they only assessed one signal, PPG. We proposed a model involving two signals, BCG and IPG with better performance than those of other methods listed in Table 3.

Liu et al. introduced a cuffless BP measurement method utilizing a commercial weight-fat scale (23, 28). They devised two measurement techniques to extract PTT: BCG plus PPG, and BCG plus IPG. The PCCs for SBP and DBP estimations using BCG and PPG signals were 0.778 and 0.533, respectively. However, the PCCs for SBP and DBP estimations using BCG and IPG signals were lower at 0.754 and 0.532, respectively. The performance of the BCG plus PPG method surpassed that of the BCG plus IPG method, primarily due to the superior stability of PPG compared to IPG, a challenging issue to address. Additionally, in these studies, the quality of BCG, PPG, and IPG signals was determined manually, making it difficult to assess the quantitative and qualitative performance of the two methods. In this study, we introduced a novel approach to define the quality of BCG and IPG pulses based on the percentage error of PAT2_ECG_ _+_ _PPG_ and PTT2_BCG_ _+_ _IPG_ for each beat. All BCG and IPG pulses within a segment had to exhibit good quality to be considered for analysis. Moreover, we used ML technique to improve the accuracy. Subsequently, the calibration-based and calibration-free PTT parameters, and mHR in this segment were utilized to estimate BP. Consequently, the PCCs and MADs for SBP and DBP estimated by XGBoost reached 0.95 and 0.94, and 3.35 and 2.67 mmHg, respectively, representing a significant improvement over previous method (28).

Previous studies have employed ML techniques to directly utilize PPG and ECG signals for BP estimation, demonstrating superior performance compared to linear regression methods (42, 51). In some instances, only temporal and spatial parameters of PPG pulses were utilized for BP estimation (42). This approach was feasible due to the high quality of PPG and ECG signals. However, in our study, the quality of BCG and IPG signals was lower than those of ECG and PPG signals. Traditionally PAT and HR parameters could be employed for BP estimation, but PTT parameters of BCG and IPG signals are more unstable than PAT of ECG and PPG signals. As a result, we should not only use the calibration-free PTT parameters, mPTT1 and mPTT2, but also the calibration-based PTT parameters, mPTT1_SYS_, mPTT1_DIA_, mPTT2_SYS_, and mPTT2_DIA_. The calibration-based PTT parameters were normalized by the resting BP of subjects. Newman and Greenwald elucidated the relationship between BP and pulse wave velocity based on the Moens-Korteweg equation (15). Thus, PAT can be regarded as a relative variable rather than an absolute one. This implies that calibration is unnecessary when using PAT to monitor BP change. But the calibration is necessary to monitor the absolute BP. Mukkamala et al. proposed an absolute blood pressure estimation, where PAT should be calibrated using appropriate means (52). In this study, we used the method of permutation feature importance to evaluate the significance of all PTT parameters for BP estimation. PTT2_SYS_ and PTT2_DIA_ have the highest scores of 1.44 and 0.92, respectively. This result aligned well with the principles of cuffless BP measurement. Table 4 shows the performances of BP estimation with calibration-based parameters, calibration-free parameters, and all parameters, respectively. When only calibration-based parameters are used to estimate SBP, PCC and MAE by XGBoost model are 0.757 and 7.4 mm Hg, and 0.753 and 7.1 mm Hg by RF model. For DBP estimation, PCC and MAE by XGBoost model are 0.736 and 5.2 mm Hg, and 0.739 and 5.1 mm Hg by RF model. When calibration-free parameters are used to estimate BP, PCC and MAE by XGBoost model are 0.834 and 6.0 mm Hg, and 0.826 and 5.8 mm Hg by RF model. For DBP estimation, PCC and MAE by XGBoost model are 0.822 and 4.4 mm Hg, and 0.833 and 4.2 mm Hg by RF model. These results are all lower than them with calibration-based and calibration-free parameters.

**Table 4:** SBP and DBP estimations use the calibration-based parameters, calibration-free parameters, and all parameters, respectively.

	Parameter	Model		PCC	MAE (mm Hg)
Calibration-based	PTT1_SYS_ PTT1_DIA_ PTT2_SYS_ PTT2_DIA_	XGBoost	SBP	0.757	7.381
			DBP	0.736	5.190
		RF	SBP	0.753	7.081
			DBP	0.739	5.078
Calibration-free	mPTT1 mPTT2 mHR	XGBoost	SBP	0.834	6.008
			DBP	0.822	4.350
		RF	SBP	0.826	5.814
			DBP	0.833	4.173
All	mPTT1 mPTT2 mHR PTT1_SYS_ PTT1_DIA_ PTT2_SYS_ PTT2_DIA_	XGBoost	SBP	0.959	3.377
			DBP	0.936	2.661
		RF	SBP	0.947	3.542
			DBP	0.940	2.575

Both RF and XGBoost models are decision-tree based methods, resulting in very similar PCCs and MAD values. For five-fold cross-validation, PCCs for SBP and DBP were 0.947 ± 0.010 and 0.940 ± 0.007 for RF model, and 0.953 ± 0.007 and 0.935 ± 0.007 for XGBoost model. MADs for SBP and DBP were 3.54 ± 0.34 and 2.57 ± 0.17 mmHg for RF model, and 3.35 ± 0.28 and 2.67 ± 0.15 mmHg for XGBoost model. RF is an ensemble learning method that employs bagging, or bootstrap aggregating, with decision tree algorithms. It creates multiple decision trees during training, each generated from a random subset of the data. On the other hand, XGBoost is also an ensemble technique, but it builds trees sequentially, with each new tree aiming to correct the errors made by the previous ones. One advantage of XGBoost is its smaller model size, but its drawback lies in longer training times. In our study, the sizes of RF and XGBoost were 5.04 and 0.31 Mbytes, respectively, with training times of 111 and 7 µs. Therefore, if the proposed method is to be embedded in a microcontroller, the XGBoost model would be a preferable choice.

Table 5 presents a comparative analysis of our method with other studies that utilized either ECG or BCG as the proximal reference, and IPG or PPG as the distal reference for cuffless blood pressure measurement. The root mean square error (RMSE), MAD, PCC are the metrics. Notably, the proposed method exclusively relied on BCG and IPG data obtained from the weight-fat scale and demonstrated superior performance compared to previous studies. Our proposed method has the best accuracy, 0.953 ± 0.007 and 0.935 ± 0.007 of PCCs, and 3.54 ± 0.34 and 2.57 ± 0.17 mmHg of MADs for SBP and DBP estimations. In this study, there are some limitations. First, reference BPs were not directly measured by the invasive continuous BP monitor. The reference BP was measured only once per minute using a digital sphygmomanometer. Subsequently, the BPs of beats within that minute were estimated through linear interpolation, as we hypothesized that BP would change linearly over time. Second, only 17 young health subjects participated in this experiment. The number of subjects is smaller than the requirement of the standard, 85 subjects. Third, the samples of hypertension categories were produced by the exercise stress, did not collect the BP from hypertension patients. Forth, the accuracy of the proposed method may be compromised for individuals with conditions such as sarcopenia or Parkinson's disease, because both BCG and IPG measurements are sensitive to body movements. Furthermore, our proposed BP measurement requires users to stand on a commercial weight-fat scale. Therefore, individuals unable to stand for at least 30 s may find this method unsuitable. Nonetheless, a significant advancement of this study lies in using artificial intelligence techniques for cuffless BP estimation.

**Table 5:** Comparative result of various methods using the ECG or BCG as the proximal reference, and IPG or PPG as the distal reference for the cuffless blood pressure measurement.

Reference	PAT/PTT Signals (sensor placement)	*PCC*	
(18)	BCG (foot) and BPW (finger)	SBP: 0.70 DBP: 0.66	NA
(19)	BCG (foot) and PPG (foot) plus interval and amplitude of BCG	SBP: 0.8 DBP:0.78	RMSE SBP: 7.3 ± 0.6 mmHg DBP: 5.7 ± 0.4 mmHg
(20)	BCG (Chair)	SBP: 0.755 DBP: 0.532	MAD SBP: 4.48 mmHg DBP: 3.84 mmHg
(21)	BCG (foot) and PPG (foot)	NA	RMSE SBP: 11.8 ± 1.6 mmHg DBP: 7.6 ± 0.5 mmHg,
(22)	BCG (hand) and ECG	SBP: 0.81 DBP: 0.80	NA
(26)	IPG (wrist) and PPG (finger)	SBP: 0.88 ± 0.07 DBP: 0.88 ± 0.06	RMSE SBP: 8.47 ± 0.91 mmHg DBP: 5.02 ± 0.73 mmHg
(27)	ECG and IPG (arm)	SBP: 0.700 DBP: 0.450	NA
(23)	BCG (foot) and PPG (foot)	SBP: 0.775 DBP: 0.532	RMSE SBP: 6.7 ± 1.6 mmHg DBP: 4.8 ± 1.47 mmHg
(28)	BCG (foot) and IPG (foot)	SBP: 0.754 DBP: 0.533	RMSE SBP: 7.3 ± 2.1 mmHg DBP: 4.5 ± 1.8 mmHg
Proposed	BCG (foot) and IPG (foot)	SBP: 0.953 ± 0.007 DBP: 0.935 ± 0.007	MAD SBP: 3.54 ± 0.34 mmHg DBP: 2.57 ± 0.17 mmHg

## 5 Conclusions

Liu et al. proposed a cuffless BP measurement using BCG and IPG signals obtained while a user stood on a commercial weight-fat scale (28). However, BCG and IPG signals are highly susceptible to motion artifacts. In this study, 17 young health subjects participated the experiment, whose BP were stressed by the exercise. The reference BP for each beat was made by the linear interpolation. The total samples included the good quality samples of 2,262 and poor quality samples of 20,358. The we employed a stacking 1D CNN + GRU model to identify the quality of synchronous BCG and IPG, achieving an accuracy of 98.9%. Furthermore, we replaced the linear regression method with ML models. We examined the calibration-based and calibration-free parameters—mPTT2, PTT2_SYS_, PTT2_DIA_, mPTT1, PTT1_SYS_, PTT1_DIA,_ and mHR—for XGBoost model under a five-fold cross-validation, which demonstrated excellent performance with PCCs for SBP and DBP 0.953 ± 0.007 and 0.935 ± 0.007, and MADs for SBP and DBP 3.35 ± 0.28 and 2.67 ± 0.15 mmHg, respectively. Thus, our study has enhanced the performance of cuffless BP measurement using BCG and IPG signals. In the future validation, a more diverse and represented population should be addressed. Finally, the advancement of this study holds promise for convenient BP monitoring in daily life, facilitating mobile health (mHealth) management in the future.

## Data Availability

The datasets presented in this article are not readily available because the dataset is unavailable due to the restriction of IRB. Requests to access the datasets should be directed to shliu@cyut.edu.tw.
